# Macrophages During the Fibrotic Process: M2 as Friend and Foe

**DOI:** 10.3389/fimmu.2015.00602

**Published:** 2015-11-25

**Authors:** Tarcio Teodoro Braga, Juan Sebastian Henao Agudelo, Niels Olsen Saraiva Camara

**Affiliations:** ^1^Immunology Department, University of São Paulo, São Paulo, Brazil; ^2^Nephrology Division, Medicine Department, Federal University of São Paulo, São Paulo, Brazil; ^3^Renal Physiology Laboratory, Faculty of Medicine, University of São Paulo, São Paulo, Brazil

**Keywords:** polarization, macrophage activation, cell metabolism, myofibroblasts, fibrosis

## Abstract

Macrophages play essential activities in homeostasis maintenance during different organism’s conditions. They may be polarized according to various stimuli, which subsequently subdivide them into distinct populations. Macrophages with inflammatory activity function mainly during pathological context, while those with regulatory activity control inflammation and also remodel the repairing process. Here, we propose to review and to present a concise discuss on the role of different components during tissue repair, including those related to innate immune receptors and metabolic modifications. The scar formation is directly related to the degree of inflammation, but also with the appearance of M2 macrophages. In spite of greater numbers of macrophages in the fibrotic phase, regulatory macrophages present some characteristics related to promotion of fibrosis but also with the control of scar formation. These regulatory macrophages present an oxidative metabolism, and differ from the initial inflammatory macrophages, which in turn, present a glycolytic characteristic, which allow regulatory ones to optimize the oxygen consumption and minimizing their ROS production. We will emphasize the difference in macrophage subpopulations and the origin and plasticity of these cells during fibrotic processes.

## Introduction

Macrophages are cells of the innate immune system highly heterogeneous, involved in the primary response to microorganisms, in inflammatory responses, homeostasis, and tissue regeneration (1). Several evidences show that initial infiltration of macrophages culminates with pro-inflammatory cytokines and reactive oxygen species (ROS) production, which exacerbates inflammatory diseases such as diabetes mellitus, kidney disease, heart, and liver disease. Conversely, macrophages in the later phase of diseases have been associated with release of anti-inflammatory molecule and growth factors, which attenuate inflammation and promote tissue regeneration (2). However, there are macrophage dysfunction, which can impair the proper regenerative process, and otherwise, promote the development of fibrosis, deposition of type I and III collagen, and myofibroblasts activation. Emerging evidence demonstrates that both inflammatory and regulatory macrophages may participate in the pro-fibrotic processes, and this event may be dependent on the macrophage origin and the intrinsic aspects of the pathology (3, 4). Below, we will discuss the differences in macrophage subpopulations characteristics and their ontogeny with emphasis in the fibrotic process.

## Ontogeny of Macrophages

Since the description of macrophages in 1888 by the renowned scientist Elie Metchnikoff (5) considerable accumulating knowledge about their biology, development, and origin were generated, re-evaluated, and placed on discussion as a result of advances in biology technology such as conditional deletion and colored-labeled-monocytes, that unquestionably enable us to better understand these cells (6–9). One example is that for years we assumed that all resident macrophages come from circulating monocytes derived from a single myeloid precursor in bone marrow (7). However, nowadays we know resident macrophages are heterogeneous cells that can develop from different sources, including embryonic progenitor cells, bone marrow hematopoietic cells or local proliferation (6–9).

Embryonic hematopoiesis begins on the eighth day after conception in the yolk sac (10, 11). Progenitors migrate to fetal liver to establish a temporary hematopoiesis (7, 12). Macrophages with embryonic origins may be regulated by CSF1R and their corresponding ligands IL-34 and CSF1 (13–15). Studies on CSF1R ablation verified CSF1R is important for the generation of resident macrophages once deletion of this receptor compromises the development of resident macrophages in brain, bone, skin among other tissue (15).

**Figure 1:**
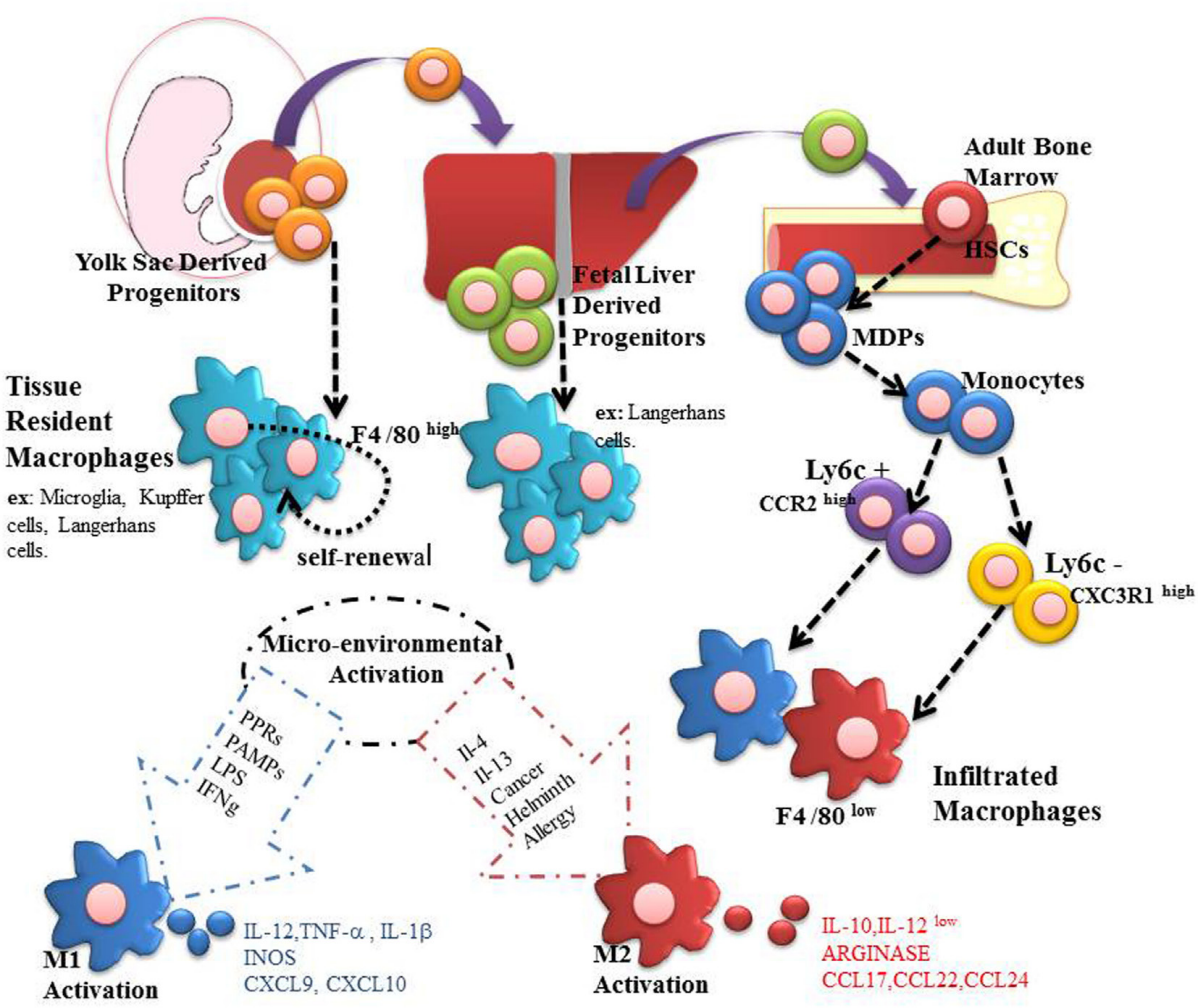
**Macrophages are present in all mammalian tissues, contributing to homeostasis and organ disease**. Most tissue macrophages have an embryonic origin, and they are not fully derived from circulating monocytes. From embryonic day 6.5–8.5, resident macrophages can be generated in yolk sac. These macrophages can be identified as being regulated by CSF1R, and they are independent of the factor myb. Subsequently, during day E 8.5 to E 10.5 hematopoietic stem cells (HSCs)-derived aorta-gonad-mesonephros can migrate to fetal liver and establish a temporary hematopoiesis, giving rise, for example, to Langerhans cells and alveolar macrophages. In addition, resident macrophages derived from fetal liver may originate both HSCs precursors and mature erythro-myeloid cells. Finally, during the perinatal period, HSCs migrate to the bone marrow to establish itself a definitive place of hematopoiesis that will last until the adulthood. On this point, they are produced as Ly6C^+^CCR2^high^ and Ly6C^−^CX3CR1^high^ monocytes capable of infiltrating organs and differentiate into macrophages. Both infiltrating and residents macrophages can be polarized to M1 and M2 according to the microenvironment stimuli.

Monocytes derived from bone marrow myeloid progenitors also give rise to both dendritic cells and macrophages. Two different types of monocytes are described in mice: Ly6C^+^CCR2^high^ and Ly6C^−^CX3CR1^high^. Ly6C^+^CCR2^high^ are called “inflammatory” monocytes and are considered to be recruited to inflamed lymph nodes and tissues where typically differentiate into DC or inflammatory macrophages. In contrast, Ly6C^−^CX3CR1^high^ present low CCR2 expression, are smaller and are known as “resident-monocyte,” responsible by surveillance in homeostatic conditions, an essential task to accomplish the cleaning oxidized lipids, dead cells, and possible pathogens (16–18). Besides, these cells are also related to reduce inflammation and promotion of tissue repair (16). A schematic origin of macrophage is shown in Figure 1.

## Macrophage Polarization

Resident and infiltrating macrophages may be polarized according to the microenvironment stimuli (6, 8). They may be considered M1, also known as classical or pro-inflammatory, and M2 also known as alternative macrophages, but with intermediate states of activation (19, 20).

Classically activation is acquired in presence of IFN-γ, derived from natural killer cells and Th1 lymphocytes, and LPS from pathogens. Such activation increases the phagocytic capacity of macrophages along with the expression of class II MHC and costimulatory molecules such as CD80/CD86 (21). This biological event makes the macrophage a cell specialized to present antigens, along with the production of inflammatory cytokines (TNF-α, IL-12, and IL-23), besides recruiting Th1 and Th17 lymphocytes. Consequently, the adaptive immune system maintains activation of macrophages in order to provide a stable defense against any pathogen. The role of M1 macrophages is associated with microbicide capacity, antigen presentation, antitumor activity, and they are related to inflammatory diseases (2, 21). M1 macrophages also express ROS and chemokines such as CCR7, CXCL9, and CXCL10 (2, 22).

M2 macrophages, in turn, present different properties, sometimes opposite, to M1 macrophages. They secrete anti-inflammatory factors, which help to diminish the inflammation (2, 20). The polarization of M2 cells is mainly promoted by Th2 cytokines such as IL-4 and IL-13. The profile of chemokines and cytokines are also different between both cases. M2 macrophages produce chemokines that recruit Th2 lymphocytes and T regulatory cells such as CCL17, CCL22, and CCL24 (23). Other features that characterize M2 macrophages are the expression of Arg1, Ym1, and Fizz, secretion of angiogenic factors such as IL-8, VEGF, and EGF4, increased mannose receptor (CD206), besides reduced expression of pro-inflammatory cytokines and ROS. M2 macrophages carry out the clearance of apoptotic cells, combat intestinal parasites, stimulate tumor growth, and promote the regeneration of organs (24, 25).

## The Role of Macrophages in the Progression of Fibrosis

### Defining Fibrosis

The repair tissue damage is a fundamental biological process that allows the orderly replacement of damaged or dead cells due to some injury, an essential mechanism for survival. The damage tissue can result from various stimuli, acute or chronic, including infections, autoimmune reactions, mechanical injury, or any stimulation of the immune response. The repair process typically involves two distinct stages: a regenerative phase, in which the damaged cells are replaced by cells of the same type without bringing any evidence of harm; and a phase called fibroplasia or more commonly called fibrosis, in which connective tissue replaces normal parenchymal tissue. Although initially beneficial, the healing process becomes pathological when it becomes continuous, resulting in substantial remodeling of the ECM and formation of permanent scar. In some cases, this can lead to organ failure (26).

Unlike acute inflammatory reactions that are characterized by fast vascular changes, edema, and neutrophil infiltration, fibrosis usually originates from chronic inflammatory responses, defined as a response that persists for several weeks or months, and which inflammation and tissue destruction process repair occur simultaneously. Although different etiologies and clinical distinction, most fibrotic disorders have in common a persistent inflammation which maintains production of growth factors, proteolytic enzymes, angiogenic factors, and pro-fibrotic cytokines, which together stimulate the deposition of connective tissue elements remodel or progressively destroy normal tissue architecture (27, 28).

Irrelevant of the initial cause, the development of interstitial fibrosis is characterized by the appearance of activated fibroblasts, positive for α-smooth muscle actin (α-SMA), also called myofibroblasts. In renal parenchyma, the deposition of ECM products is largely attributed to these cells (29).

### Myofibroblasts as Effector Cells in Fibrosis

Myofibroblasts are recognized as the effector cells of fibrogenesis (30). These cells are recognized by synthesizing large amounts of ECM, a substance which is mainly comprised of fibers of type I and III collagen, fibronectin, laminin, and other basal membrane proteins that are the source of scar tissue (31, 32). In addition, myofibroblasts are characterized by generating contractility, and distort the architecture organs, a property that is due to the expression of smooth muscle proteins as α-SMA (33). It has been identified at least three different sources for myofibroblasts (34). The first origin is related to the activation of local stromal cells such as fibroblasts and pericytes in the presence of pro-fibrotic factors (35). The second myofibroblasts source is from circulating fibrocytes. These cells originate in the bone marrow and express markers such as CD34, CD45RO, 25F9, S100A8/A9, and type 1 collagen (36). They can be recruited by inflammatory chemokines, and its importance is related to the role they have in lung, skin, heart, liver, and kidney fibrosis process (37). Other sources of myofibroblasts are epithelium or endothelium to mesenchymal transition (EMT and EndoMT), a reported process that occur in tubular cells in the presence of TGF-β in which such cells may adopt mesenchymal characteristics (38, 39). During EMT and EndoMT, renal tubular cells lose their phenotype and thus transdifferentiate into myofibroblast cells expressing α-SMA and type I collagen. The EMT/EndoMT process involves four key events: (1) loss of epithelial adhesion properties, (2) new α-sMA expression and actin reorganization, (3) increased permeability of the tubular basement membrane, and (4) increased migration and invasion ability (40).

TGF-β is the only factor described as participating in the four events of EMT and two molecules: hepatocytes growth factor (HGF) (41) and bone morphogenic protein-7 (BMP-7) (42) have been demonstrated as being capable of reversing the process of EMT due to inhibition of TGF-β and hence decreasing renal fibrosis.

### Macrophages and Fibrosis

Since embryonic stages, it has been shown that CSF1R^+^ macrophages participate in the homeostasis and architectural remodeling of tissue (43). However, it has also been shown that the degree and severity of damage and fibrosis correlates with infiltrating macrophages (44). Depletion of resident macrophages by clodronate or CCL2 blockade improves kidney injury and reduce the pro-fibrotic process (45, 46). Interestingly, Nishida et al. showed that there are apparently infiltrating macrophages with opposing functions, once angiotensin II type 1 receptor (AGTR1^+^) macrophages have an anti-fibrotic role. In fact, it was observed that AGTR1^−/−^ animals have a more pronounced fibrosis (47). This suggests that there are diverse populations of macrophages that infiltrate the kidney, with pro- and anti-fibrotic capacities which could be related to the time the injury happens.

M1 macrophages are known to predominate during the onset of injury (48–50). They release pro-inflammatory cytokines that exacerbate the injury, amplify the inflammatory response and contribute to myofibroblasts proliferation and recruitment of fibrocytes (4, 32). M1 have been associated with the release of metalloproteinases that degrade ECM and promote EMT/EndoMT (51). It was shown that blocking MMP-9 or MMP-2 results in reduction of fibrosis in the UUO model of disease (52). Oppositely, Lutz et al., have demonstrated that inhibition of MMP-2 in chronic allograft nephropathy results in a more severe fibrosis (53), which suggests that MMPs are also important enzymes for the control of fibrosis and scarring area limitation. Macrophage secretion of MMP-9, MMP-12 and MMP-13 in the liver is related to ECM degradation and resolution of fibrosis (54, 55). Also, it has been identified a Ly6C^low^ macrophage population that secrete MMPs and have anti-fibrotic role in the liver (56). However, by transcriptional analysis, such macrophage population could not be classified as M1 or M2. In the liver, as in the kidney, macrophages have an important role in fibrosis progression. For example, there is strong evidence showing that Kupffer cells activate hepatic stellate cells to promote their transdifferentiation into myofibroblasts (57). These cells are the main source of ECM in the liver and they are responsible for the progression of cirrhosis.

When the acute phase of inflammation finishes, Th2 cytokines are produced to promote the polarization and recruitment of M2 macrophage (58). Added to this, apoptotic cells are recognized and phagocytosed by macrophages M1, an event that also promotes macrophage alternative activation (59). M2 macrophages are intended to create an anti-inflammatory environment and promote healing and regeneration of wounds. However, when the lesion is persistent, M2 macrophages take an important pro-fibrotic role and these cell population are known to secreting large amounts of pro-fibrotic factors such as TGF-β and Galactin-3 (60). The latter is a protein that is widely associated with cardiac fibrosis and atrial fibrillation (61). Preclinical studies have shown that infusion of recombinant galectin-3 activates cardiac fibroblast proliferation, leading to ventricular dysfunction (62). Furthermore, it has been observed that patients with paroxysmal atrial fibrillation have elevated levels of galectin-3 (63). Therefore, some authors believe that galectin-3 could act as heart failure and fibrosis biomarker. Furthermore, Braga et al. showed that in the absence of IL-4, mice underwent UUO are associated with improved parameters and decreased renal fibrosis (64).

### M1 and M2 in the Context of Myofibroblasts Activation

There are growing evidences showing the relationship between macrophages and myofibroblasts activation during inflammation by different ways (50). M1 macrophages generate cytokines that activate myofibroblasts, either by the production of pro-inflammatory cytokines such as TNF-α and IL-1β, or chemokine production, such as CCL2 that assist in fibrocytes recruitment (65). Fibrocytes can migrate to the site of the inflammation through the expression of receptors as CCR2, CCR3, CCR7, and CXCR4 (37, 66).

**Figure 2:**
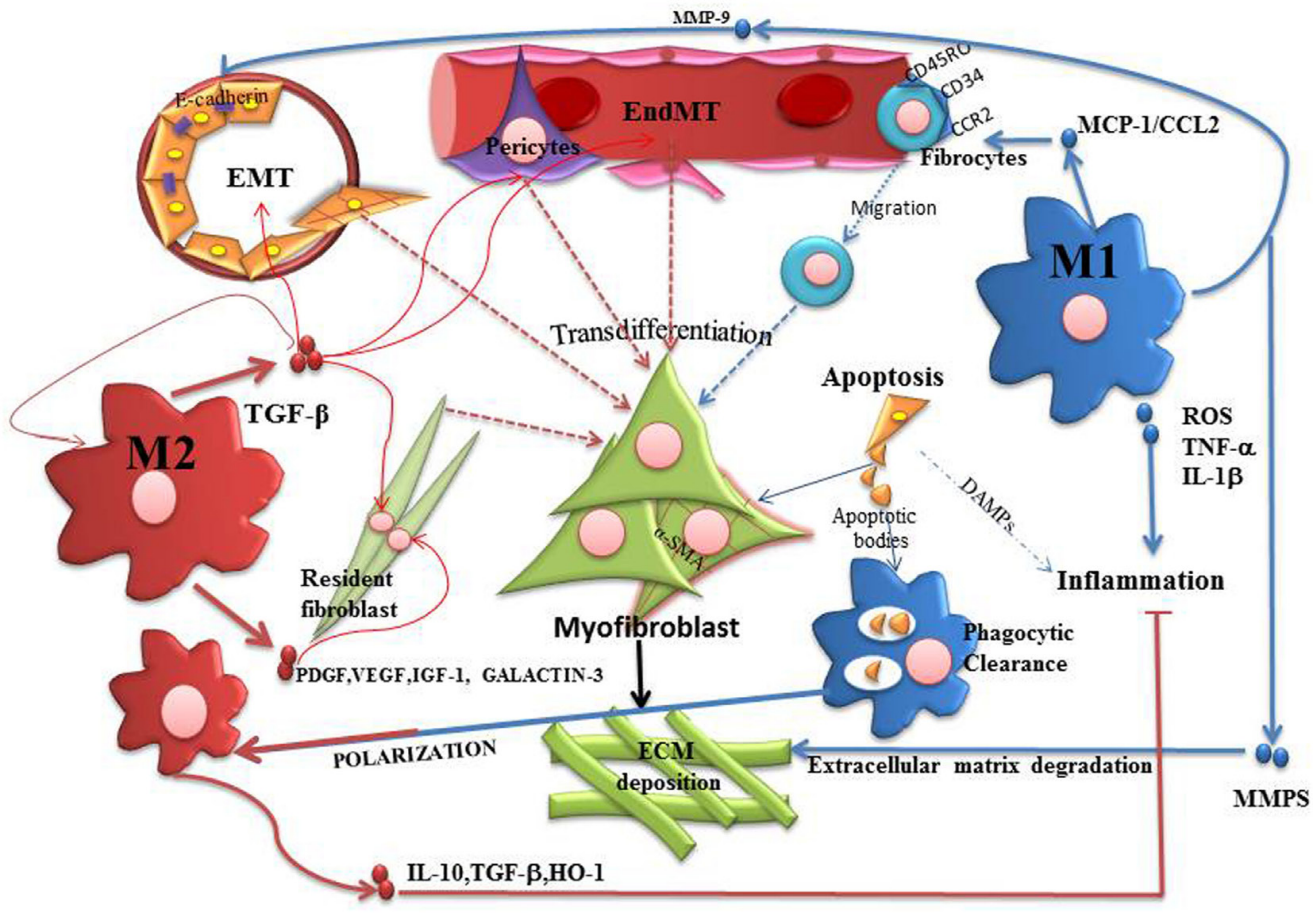
**Participation of M1 and M2 macrophages in the process of fibrosis**. The activation of myofibroblasts is a physiological process generated in order to repair and restore tissue homeostasis. However, if the insult persists, fibrosis progress with proliferation of myofibroblasts and deposition of ECM which replaces functional tissue, leading to scar tissue formation. In this context, M1 macrophages represent the starting point of pro-fibrotic process. Therefore, M1 releases pro-inflammatory cytokines and chemokines that indirectly promote the proliferation of myofibroblasts. M1 can release CCL2, assisting in the recruitment of fibrocytes and also MMP-9, that promotes EMT/EndoMT. M1 has also been associated with an anti-fibrotic effect by releasing MMPs that degrade ECM. M2 macrophages can be generated by phagocytosis of apoptotic bodies or Th2 cytokines stimulation. M2 is initially anti-inflammatory cells through the release of IL10, arginase, TGFβ and HO-1. But when the damage persists, M2 activation leads to EMT/EndoMT as well as proliferation of fibrocytes due to the release of several growth factors. In this sense, macrophage modulation is the central axis of the exacerbation or control of fibrosis.

M2 macrophages contribute to the control of inflammatory process through the release of IL10, arginase, TGF-β and HO-1, a process which promotes controlled wound healing and tissue regeneration (67, 68). However, the healing process depends on whether the initial insult persists or not (69). In this sense, if the insult persists, chronic activation of M2 leads to an opposite effect. M2 can activate resident fibroblasts through the release of TGF-β, PDGF, VEGF, IGF-1, and Galactin-3 (50, 57, 70). This evidence demonstrates that the exacerbation of fibrosis could depend on the type of macrophage polarization and persistence of the inflammatory insult, as shown in Figure 2.

### Macrophage Metabolism Regulation in Fibrosis

Recently, a large amount of data has been coming in focus concerning metabolism and macrophage plasticity (71–73). We know M1 macrophages present an glycolytic cellular metabolism (74). It has been shown LPS, in an M1 polarization context, induces the transcriptional factor HIF-1α, which, in turn, transcriptionally couples glycolytic metabolism to macrophages’ inflammatory and microbicidal programs (74). HIF-1α is stabilized by succinate, an effect that is inhibited by 2-deoxyglucose, a glycolytic pathway inhibitor (75). A metabolomic screen of LPS-stimulated macrophages revealed not only the expected activation of the Warburg effect but also an unexpected accumulation of intermediates of the tricyclic acid cycle, in particular succinate (75, 76). In M1 macrophages, it was also identified a metabolic break at the enzyme that converts isocitrate to α-ketoglutarate, providing mechanistic explanation for tricyclic acid cycle fragmentation (76).

On the other hand, M2 polarization was found to activate glutamine catabolism (76). Given that M2 macrophage activation and chronic diseases are energetically demanding, both in terms of intensity and duration, Vats et al., demonstrated that distinct substrates and pathways might meet the metabolic demands of M2 (77). Microarray analysis of M2 revealed that genes important in fatty acid oxidation were preferentially expressed in such cells. Metabolic studies further verified that M2 present increased mitochondrial amount and function. Accordingly, inhibition of oxidative phosphorylation by metabolic inhibitors dramatically diminished the expression of M2 markers (77). It is also known that IL-4 and IL-13 induce oxidative metabolism by inhibiting mTOR, via activation of its upstream negative regulators TSC1 and TSC2 (78). Inhibition of mTOR can also lead to a decrease in HIF-1α levels, and therefore could result in reduced HIF-1α-dependent glycolytic and inflammatory gene expression (79).

There is a clear distinction in metabolism between macrophage subtypes, otherwise, the relevance of these observations and the implications for fibrosis are not fully understood. It is known pulmonary fibrosis development is related to mutations in maternally inherited mtDNA encoding for key genes of mitochondrial energy-generating oxidative phosphorylation, rather than Mendelian nuclear genetic principles (80, 81). Mitochondrial ROS are also responsible by death of alveolar epithelial cells in the context of fibrosis originated from fibrogenic dusts, such as asbestos and silica (82). Also, liver kinase B1 (*Lkb1*), an upstream regulator of fatty acid metabolism, has been implicated in chronic kidney disease (CKD) development (83). Loss of *Lkb1* impaired metabolic signaling and caused intracellular lipid accumulation, impaired fatty acid oxidation, and decreased glycolysis compared to control cells. Subcellular analyses of the mutant cells also identified a distorted mitochondrial structure, which negatively impacted upon cellular ATP content (83). Besides fatty acid, glucose metabolism has been implicated in CKD. High glucose concentrations may play important role in fibrosis development once leads to up-regulation expression of TGFβ, Smad3, Smad7, and CTGF (84).

However, much is expected in order to correlate macrophage metabolism and fibrosis formation. We still do not understand the scar formation in the context of drugs capable to modulate the metabolism in cells. It is known that chronic ethanol consumption disturbs several hepatic enzymes, including those related to cellular metabolism, such as PGC-1α (85), in a cirrhosis model of disease, meanwhile new studies in fibrotic models that do not are related to metabolites ingestions are needed.

## Conclusion

Macrophages represent a heterogeneous cell population that can develop from different sources. M1 macrophages are associated with pro-inflammatory functions, and an exacerbation of tissue inflammation initiates the pro-fibrotic process (69). In this direction, M1 activates myofibroblasts through the release of MMPs that promote EMT/EndoMT and fibrocytes recruitment through CCL2 secretion. On the other hand, M2 macrophages have anti-inflammatory properties due to the ability to secrete IL-10, arginase, TGFβ, and HO-1 (65, 68). In this point of view, M2 becomes friend of the tissue repairing. However, when the insult is not controlled and there is a persistent activity of M2 macrophages, these cells act as an enemy for tissue homeostasis. Excessive M2 macrophage activation leads to the continuous production TGFβ and growth factors that promote proliferation of myofibroblasts, activation of EMT/EndoMT and ECM deposition (34). In this scenario, M2 represents a break point between wound healing and exacerbation of pro-fibrotic process. Recently, much has been studied about macrophages metabolism. We know, for example, that pro-inflammatory cells present a glycolytic metabolism while anti-inflammatory ones are characterized by an oxidative metabolism. Otherwise, more studies are needed in order to identify macrophages components responsible by fibrosis triggering and different intervention manners in fibrotic process.

## Conflict of Interest Statement

The authors declare that the research was conducted in the absence of any commercial or financial relationships that could be construed as a potential conflict of interest.

## References

[1] Gordon S Taylor PR Monocyte and macrophage heterogeneity. Nat Rev Immunol (2005) 5(12):953–64. 10.1038/nri1733 16322748

[2] Sica A Mantovani A. Macrophage plasticity and polarization: in vivo veritas. J Clin Invest (2012) 122(3):787–95. 10.1172/JCI59643 22378047 PMC3287223

[3] Duffield JS. Macrophages and immunologic inflammation of the kidney. Semin Nephrol (2010) 30(3):234–54. 10.1016/j.semnephrol.2010.03.003 20620669 PMC2922007

[4] Wynn TA Barron L. Macrophages: master regulators of inflammation and fibrosis. Semin Liver Dis (2010) 30(3):245–57. 10.1055/s-0030-1255354 20665377 PMC2924662

[5] Cavaillon JM. The historical milestones in the understanding of leukocyte biology initiated by Elie Metchnikoff. J Leukoc Biol (2011) 90(3):413–24. 10.1189/jlb.0211094 21628329

[6] Geissmann F Manz MG Jung S Sieweke MH Merad M Ley K. Development of monocytes, macrophages, and dendritic cells. Science (2010) 327(5966):656–61. 10.1126/science.1178331 20133564 PMC2887389

[7] Wynn TA Chawla A Macrophage biology in development, homeostasis and disease. Nature (2013) 496(7446):445–55. 10.1038/nature12034 23619691 PMC3725458

[8] Dey A Allen J Hankey-Giblin PA. Ontogeny and polarization of macrophages in inflammation: blood monocytes versus tissue macrophages. Front Immunol (2014) 5:683. 10.3389/fimmu.2014.00683 25657646 PMC4303141

[9] Cassado Ados A D’Império Lima MR Bortoluci KR. Revisiting mouse peritoneal macrophages: heterogeneity, development, and function. Front Immunol (2015) 6:225. 10.3389/fimmu.2015.00225 26042120 PMC4437037

[10] Lichanska AM Hume DA Origins and functions of phagocytes in the embryo. Exp Hematol (2000) 28(6):601–11. 10.1016/S0301-472X(00)00157-0 10880746

[11] Shepard JL Zon LI. Developmental derivation of embryonic and adult macrophages. Curr Opin Hematol (2000) 7(1):3–8. 10.1097/00062752-200001000-00002 10608497

[12] Schulz C Gomez Perdiguero E A lineage of myeloid cells independent of Myb and hematopoietic stem cells. Science (2012) 336(6077):86–90. 10.1126/science.1219179 22442384

[13] Chitu V Stanley ER Colony-stimulating factor-1 in immunity and inflammation. Curr Opin Immunol (2006) 18(1):39–48. 10.1016/j.coi.2005.11.006 16337366

[14] Wei S Nandi S Chitu V Yeung YG Yu W Huang M Functional overlap but differential expression of CSF-1 and IL-34 in their CSF-1 receptor-mediated regulation of myeloid cells. J Leukoc Biol (2010) 88(3):495–505. 10.1189/jlb.1209822 20504948 PMC2924605

[15] Gomez Perdiguero E Klapproth K Schulz C Busch K Azzoni E Crozet L Tissue-resident macrophages originate from yolk-sac-derived erythro-myeloid progenitors. Nature (2015) 518(7540):547–51. 10.1038/nature13989 25470051 PMC5997177

[16] Geissmann F Jung S Littman DR. Blood monocytes consist of two principal subsets with distinct migratory properties. Immunity (2003) 19(1):71–82. 10.1016/S1074-7613(03)00174-2 12871640

[17] Auffray C Fogg D Garfa M Elain G Join-Lambert O Kayal S Monitoring of blood vessels and tissues by a population of monocytes with patrolling behavior. Science (2007) 317(5838):666–70. 10.1126/science.1142883 17673663

[18] Auffray C Sieweke MH Geissmann F. Blood monocytes: development, heterogeneity, and relationship with dendritic cells. Annu Rev Immunol (2009) 27:669–92. 10.1146/annurev.immunol.021908.132557 19132917

[19] Mills CD Kincaid K Alt JM Heilman MJ Hill AM. M-1/M-2 macrophages and the Th1/Th2 paradigm. J Immunol (2000) 164(12):6166–73. 10.4049/jimmunol.164.12.6166 10843666

[20] Mosser DM Edwards JP. Exploring the full spectrum of macrophage activation. Nat Rev Immunol (2008) 8(12):958–69. 10.1038/nri2448 19029990 PMC2724991

[21] Biswas SK Mantovani A. Macrophage plasticity and interaction with lymphocyte subsets: cancer as a paradigm. Nat Immunol (2010) 11(10):889–96. 10.1038/ni.1937 20856220

[22] Mantovani A Sica A Sozzani S Allavena P Vecchi A Locati M. The chemokine system in diverse forms of macrophage activation and polarization. Trends Immunol (2004) 25(12):677–86. 10.1016/j.it.2004.09.015 15530839

[23] Martinez FO Gordon S Locati M Mantovani A. Transcriptional profiling of the human monocyte-to-macrophage differentiation and polarization: new molecules and patterns of gene expression. J Immunol (2006) 177(10):7303–11. 10.4049/jimmunol.177.10.7303 17082649

[24] Pesce J Kaviratne M Ramalingam TR Thompson RW Urban JF Jr Cheever AW The IL-21 receptor augments Th2 effector function and alternative macrophage activation. J Clin Invest (2006) 116(7):2044–55. 10.1172/JCI27727 16778988 PMC1479424

[25] Kurowska-Stolarska M Stolarski B Kewin P Murphy G Corrigan CJ Ying S IL-33 amplifies the polarization of alternatively activated macrophages that contribute to airway inflammation. J Immunol (2009) 183(10):6469–77. 10.4049/jimmunol.0901575 19841166

[26] Wynn TA Cellular and molecular mechanisms of fibrosis. J Pathol (2008) 214(2):199–210. 10.1002/path.2277 18161745 PMC2693329

[27] Tomasek JJ Gabbiani G Hinz B Chaponnier C Brown RA. Myofibroblasts and mechano-regulation of connective tissue remodelling. Nat Rev Mol Cell Biol (2002) 3(5):349–63. 10.1038/nrm809 11988769

[28] Friedman SL. Mechanisms of disease: mechanisms of hepatic fibrosis and therapeutic implications. Nat Clin Pract Gastroenterol Hepatol (2004) 1(2):98–105. 10.1038/ncpgasthep0055 16265071

[29] Liu JY Li SR Ji SX. [Effects of connective tissue growth factor antisense oligonucleotides on the cultured human keloid fibroblasts in vitro]. Zhonghua Zheng Xing Wai Ke Za Zhi (2004) 20(6):454–6. 15835807

[30] Hinz B Phan SH Thannickal VJ Galli A Bochaton-Piallat ML Gabbiani G. The myofibroblast: one function, multiple origins. Am J Pathol (2007) 170(6):1807–16. 10.2353/ajpath.2007.070112 17525249 PMC1899462

[31] Rockey DC Housset CN Friedman SL. Activation-dependent contractility of rat hepatic lipocytes in culture and in vivo. J Clin Invest (1993) 92(4):1795–804. 10.1172/JCI116769 8408632 PMC288342

[32] Tacke F Zimmermann HW. Macrophage heterogeneity in liver injury and fibrosis. J Hepatol (2014) 60(5):1090–6. 10.1016/j.jhep.2013.12.025 24412603

[33] Hinz B Celetta G Tomasek JJ Gabbiani G Chaponnier C. Alpha-smooth muscle actin expression upregulates fibroblast contractile activity. Mol Biol Cell (2001) 12(9):2730–41. 10.1091/mbc.12.9.2730 11553712 PMC59708

[34] Conway B Hughes J. Cellular orchestrators of renal fibrosis. QJM (2012) 105(7):611–5. 10.1093/qjmed/hcr235 22139500

[35] Hinz B Phan SH Thannickal VJ Prunotto M Desmoulière A Varga J Recent developments in myofibroblast biology: paradigms for connective tissue remodeling. Am J Pathol (2012) 180(4):1340–55. 10.1016/j.ajpath.2012.02.004 22387320 PMC3640252

[36] Pilling D Fan T Huang D Kaul B Gomer RH. Identification of markers that distinguish monocyte-derived fibrocytes from monocytes, macrophages, and fibroblasts. PLoS One (2009) 4(10):e7475. 10.1371/journal.pone.0007475 19834619 PMC2759556

[37] Herzog EL Bucala R. Fibrocytes in health and disease. Exp Hematol (2010) 38(7):548–56. 10.1016/j.exphem.2010.03.004 20303382 PMC3136351

[38] Forino M Torregrossa R Ceol M TGFbeta1 induces epithelial-mesenchymal transition, but not myofibroblast transdifferentiation of human kidney tubular epithelial cells in primary culture. Int J Exp Pathol (2006) 87(3):197–208. 10.1111/j.1365-2613.2006.00479.x 16709228 PMC2517360

[39] Li J Bertram JF. Review: endothelial-myofibroblast transition, a new player in diabetic renal fibrosis. Nephrology (Carlton) (2010) 15(5):507–12. 10.1111/j.1440-1797.2010.01363.x 20649869

[40] Stahl D Venetz JP Lacroix-Desmazes S Rondeau E Bonnin E Kazatchkine MD Idiopathic membranous glomerulonephritis is associated with altered patterns of self-reactive IgM and IgG antibody repertoires. Scand J Immunol (2001) 54(5):534–42. 10.1046/j.1365-3083.2001.01000.x 11696207

[41] Yang L Liu X Fu H Qiang O Huang M. [TGF beta 1 and ET-1 expression in the peripheral blood of patients with cirrhosis]. Hua Xi Yi Ke Da Xue Xue Bao (2001) 32(2):202–3. 12600085

[42] Zeisberg M Bottiglio C Kumar N Maeshima Y Strutz F Müller GA Bone morphogenic protein-7 inhibits progression of chronic renal fibrosis associated with two genetic mouse models. Am J Physiol Renal Physiol (2003) 285(6):F1060–7. 10.1152/ajprenal.00191.2002 12915382

[43] Rae F Woods K Sasmono T Campanale N Taylor D Ovchinnikov DA Characterisation and trophic functions of murine embryonic macrophages based upon the use of a Csf1r-EGFP transgene reporter. Dev Biol (2007) 308(1):232–46. 10.1016/j.ydbio.2007.05.027 17597598

[44] Eardley KS Zehnder D Quinkler M Lepenies J Bates RL Savage CO The relationship between albuminuria, MCP-1/CCL2, and interstitial macrophages in chronic kidney disease. Kidney Int (2006) 69(7):1189–97. 10.1038/sj.ki.5000212 16609683

[45] Lim JH. Radiologic findings of clonorchiasis. AJR Am J Roentgenol (1990) 155(5):1001–8. 10.2214/ajr.155.5.2120938 2120925

[46] Kitamoto K Machida Y Uchida J Izumi Y Shiota M Nakao T Effects of liposome clodronate on renal leukocyte populations and renal fibrosis in murine obstructive nephropathy. J Pharmacol Sci (2009) 111(3):285–92. 10.1254/jphs.09227FP 19893275

[47] Nishida M Fujinaka H Matsusaka T Price J Kon V Fogo AB Absence of angiotensin II type 1 receptor in bone marrow-derived cells is detrimental in the evolution of renal fibrosis. J Clin Invest (2002) 110(12):1859–68. 10.1172/JCI200215045 12488436 PMC151648

[48] Koh TJ DiPietro LA. Inflammation and wound healing: the role of the macrophage. Expert Rev Mol Med (2011) 13:e23. 10.1017/S1462399411001943 21740602 PMC3596046

[49] Mahdavian Delavary B van der Veer WM van Egmond M Niessen FB Beelen RH. Macrophages in skin injury and repair. Immunobiology (2011) 216(7):753–62. 10.1016/j.imbio.2011.01.001 21281986

[50] Lech M Anders HJ. Macrophages and fibrosis: how resident and infiltrating mononuclear phagocytes orchestrate all phases of tissue injury and repair. Biochim Biophys Acta (2013) 1832(7):989–97. 10.1016/j.bbadis.2012.12.001 23246690

[51] Cheng S Lovett DH. Gelatinase A (MMP-2) is necessary and sufficient for renal tubular cell epithelial-mesenchymal transformation. Am J Pathol (2003) 162(6):1937–49. 10.1016/S0002-9440(10)64327-1 12759250 PMC1868144

[52] Tan TK Zheng G Hsu TT Lee SR Zhang J Zhao Y Matrix metalloproteinase-9 of tubular and macrophage origin contributes to the pathogenesis of renal fibrosis via macrophage recruitment through osteopontin cleavage. Lab Invest (2013) 93(4):434–49. 10.1038/labinvest.2013.121 23358111

[53] Lutz J Yao Y Song E Antus B Hamar P Liu S Inhibition of matrix metalloproteinases during chronic allograft nephropathy in rats. Transplantation (2005) 79(6):655–61. 10.1097/01.TP.0000151644.85832.B5 15785371

[54] Fallowfield JA Mizuno M Kendall TJ Constandinou CM Benyon RC Duffield JS Scar-associated macrophages are a major source of hepatic matrix metalloproteinase-13 and facilitate the resolution of murine hepatic fibrosis. J Immunol (2007) 178(8):5288–95. 10.4049/jimmunol.178.8.5288 17404313

[55] Pellicoro A Aucott RL Ramachandran P Robson AJ Fallowfield JA Snowdon VK Elastin accumulation is regulated at the level of degradation by macrophage metalloelastase (MMP-12) during experimental liver fibrosis. Hepatology (2012) 55(6):1965–75. 10.1002/hep.25567 22223197

[56] Ramachandran P Pellicoro A Vernon MA Boulter L Aucott RL Ali A Differential Ly-6C expression identifies the recruited macrophage phenotype, which orchestrates the regression of murine liver fibrosis. Proc Natl Acad Sci U S A (2012) 109(46):E3186–95. 10.1073/pnas.1119964109 23100531 PMC3503234

[57] Pradere JP Kluwe J De Minicis S Jiao JJ Gwak GY Dapito DH Hepatic macrophages but not dendritic cells contribute to liver fibrosis by promoting the survival of activated hepatic stellate cells in mice. Hepatology (2013) 58(4):1461–73. 10.1002/hep.26429 23553591 PMC3848418

[58] Ricardo SD van Goor H Eddy AA. Macrophage diversity in renal injury and repair. J Clin Invest (2008) 118(11):3522–30. 10.1172/JCI36150 18982158 PMC2575702

[59] Fadok VA Bratton DL Konowal A Freed PW Westcott JY Henson PM. Macrophages that have ingested apoptotic cells in vitro inhibit proinflammatory cytokine production through autocrine/paracrine mechanisms involving TGF-beta, PGE2, and PAF. J Clin Invest (1998) 101(4):890–8. 10.1172/JCI1112 9466984 PMC508637

[60] Vernon MA Mylonas KJ Hughes J. Macrophages and renal fibrosis. Semin Nephrol (2010) 30(3):302–17. 10.1016/j.semnephrol.2010.03.004 20620674

[61] Boisvert R Boisclair G Picotte L. [The dental health of institutionalized elderly patients: from awareness to action]. J Can Dent Assoc (1990) 56(3):223–4. 2331630

[62] Sharma UC Pokharel S van Brakel TJ van Berlo JH Cleutjens JP Schroen B Galectin-3 marks activated macrophages in failure-prone hypertrophied hearts and contributes to cardiac dysfunction. Circulation (2004) 110(19):3121–8. 10.1161/01.CIR.0000147181.65298.4D 15520318

[63] Clementy N Piver E Galectin-3 in patients undergoing ablation of atrial fibrillation. IJC Metabolic & Endocrine (2014) 5(0):56–60. 10.1016/j.ijcme.2014.10.003

[64] Braga TT Correa-Costa M Guise YF Castoldi A de Oliveira CD Hyane MI MyD88 signaling pathway is involved in renal fibrosis by favoring a TH2 immune response and activating alternative M2 macrophages. Mol Med (2012) 18:1231–9. 10.2119/molmed.2012.00131 22777483 PMC3510298

[65] Cao Q Wang Y Harris DC. Macrophage heterogeneity, phenotypes, and roles in renal fibrosis. Kidney Int Suppl (2011) (2014) 4(1):16–9. 10.1038/kisup.2014.4 26312145 PMC4536959

[66] Phillips RJ Burdick MD Hong K Lutz MA Murray LA Xue YY Circulating fibrocytes traffic to the lungs in response to CXCL12 and mediate fibrosis. J Clin Invest (2004) 114(3):438–46. 10.1172/JCI200420997 15286810 PMC484979

[67] Nikolic-Paterson DJ Wang S Lan HY. Macrophages promote renal fibrosis through direct and indirect mechanisms. Kidney Int Suppl (2011) (2014) 4(1):34–8. 10.1038/kisup.2014.7 26312148 PMC4536961

[68] Cao Q Harris DC Macrophages in kidney injury, inflammation, and fibrosis. Physiology (Bethesda) (2015) 30(3):183–94. 10.1152/physiol.00046.2014 25933819

[69] Wynn TA Ramalingam TR. Mechanisms of fibrosis: therapeutic translation for fibrotic disease. Nat Med (2012) 18(7):1028–40. 10.1038/nm.2807 22772564 PMC3405917

[70] Lin YH Chou CH Wu XM Chang YY Hung CS Chen YH Aldosterone induced galectin-3 secretion in vitro and in vivo: from cells to humans. PLoS One (2014) 9(9):e95254. 10.1371/journal.pone.0085728 25180794 PMC4152338

[71] Ganeshan K Chawla A. Metabolic regulation of immune responses. Annu Rev Immunol (2014) 32:609–34. 10.1146/annurev-immunol-032713-120236 24655299 PMC5800786

[72] Weinberg SE Sena LA Chandel NS. Mitochondria in the regulation of innate and adaptive immunity. Immunity (2015) 42(3):406–17. 10.1016/j.immuni.2015.02.002 25786173 PMC4365295

[73] Zhu L Zhao Q Yang T Ding W Zhao Y. Cellular metabolism and macrophage functional polarization. Int Rev Immunol (2015) 34(1):82–100. 10.3109/08830185.2014.969421 25340307

[74] Kelly B O’Neill LA Metabolic reprogramming in macrophages and dendritic cells in innate immunity. Cell Res (2015) 25(7):771–84. 10.1038/cr.2015.68 26045163 PMC4493277

[75] Tannahill GM Curtis AM Adamik J Palsson-McDermott EM McGettrick AF Goel G Succinate is an inflammatory signal that induces IL-1beta through HIF-1alpha. Nature (2013) 496(7444):238–42. 10.1038/nature11986 23535595 PMC4031686

[76] Jha AK Huang SC Sergushichev A Lampropoulou V Ivanova Y Loginicheva E Network integration of parallel metabolic and transcriptional data reveals metabolic modules that regulate macrophage polarization. Immunity (2015) 42(3):419–30. 10.1016/j.immuni.2015.02.005 25786174

[77] Vats D Mukundan L Odegaard JI Zhang L Smith KL Morel CR Oxidative metabolism and PGC-1beta attenuate macrophage-mediated inflammation. Cell Metab (2006) 4(1):13–24. 10.1016/j.cmet.2006.08.006 16814729 PMC1904486

[78] Byles V Covarrubias AJ Ben-Sahra I Lamming DW Sabatini DM Manning BD The TSC-mTOR pathway regulates macrophage polarization. Nat Commun (2013) 4:2834. 10.1038/ncomms3834 24280772 PMC3876736

[79] Pawlus MR Hu CJ. Enhanceosomes as integrators of hypoxia inducible factor (HIF) and other transcription factors in the hypoxic transcriptional response. Cell Signal (2013) 25(9):1895–903. 10.1016/j.cellsig.2013.05.018 23707522 PMC3700616

[80] Wallace DC. A mitochondrial bioenergetic etiology of disease. J Clin Invest (2013) 123(4):1405–12. 10.1172/JCI61398 23543062 PMC3614529

[81] Wallace DC Chalkia D. Mitochondrial DNA genetics and the heteroplasmy conundrum in evolution and disease. Cold Spring Harb Perspect Biol (2013) 5(11):a021220. 10.1101/cshperspect.a021220 24186072 PMC3809581

[82] Kim SJ Cheresh P Jablonski RP Williams DB Kamp DW. The role of mitochondrial DNA in mediating alveolar epithelial cell apoptosis and pulmonary fibrosis. Int J Mol Sci (2015) 16(9):21486–519. 10.3390/ijms161023259 26370974 PMC4613264

[83] Han SH Malaga-Dieguez L Chinga F Kang HM Tao J Reidy K Deletion of Lkb1 in renal tubular epithelial cells leads to CKD by altering metabolism. J Am Soc Nephrol (2015). 10.1681/ASN.2014121181 26054542 PMC4731117

[84] Qiu J Tu Z Shi Y Zhang L Li Q Wang W Interference of cyclosporine on glucose metabolism: potential role in chronic transplantation kidney fibrosis. Transplant Proc (2006) 38(7):2065–8. 10.1016/j.transproceed.2006.06.047 16980001

[85] You M Jogasuria A Taylor C Wu J. Sirtuin 1 signaling and alcoholic fatty liver disease. Hepatobiliary Surg Nutr (2015) 4(2):88–100. 10.3978/j.issn.2304-3881.2014.12.06 26005675 PMC4405418

